# Decellularized Articular Cartilage Microgels as Microcarriers for Expansion of Mesenchymal Stem Cells

**DOI:** 10.3390/gels8030148

**Published:** 2022-02-27

**Authors:** Esmaiel Jabbari, Azadeh Sepahvandi, Safaa Ibrahim Kader, Victor Anthony Madormo

**Affiliations:** 1 Biomaterials and Tissue Engineering Laboratory, Department of Chemical Engineering, University of South Carolina, Columbia, SC 29208, USA; sepahvan@mailbox.sc.edu (A.S.); kaders@mailbox.sc.edu (S.I.K.); victor_madormo@med.unc.edu (V.A.M.); 2 Department of Chemistry and Biochemistry, University of South Carolina, Columbia, SC 29208, USA

**Keywords:** bovine articular cartilage, decellularization, micronization, microcarrier, mesenchymal stem cells, articular cartilage regeneration

## Abstract

Conventional microcarriers used for expansion of human mesenchymal stem cells (hMSCs) require detachment and separation of the cells from the carrier prior to use in clinical applications for regeneration of articular cartilage, and the carrier can cause undesirable phenotypic changes in the expanded cells. This work describes a novel approach to expand hMSCs on biomimetic carriers based on adult or fetal decellularized bovine articular cartilage that supports tissue regeneration without the need to detach the expanded cells from the carrier. In this approach, the fetal or adult bovine articular cartilage was minced, decellularized, freeze-dried, ground, and sieved to produce articular cartilage microgels (CMGs) in a specified size range. Next, the hMSCs were expanded on CMGs in a bioreactor in basal medium to generate hMSC-loaded CMG microgels (CMG-MSCs). Then, the CMG-MSCs were suspended in sodium alginate, injected in a mold, crosslinked with calcium chloride, and incubated in chondrogenic medium as an injectable cellular construct for regeneration of articular cartilage. The expression of chondrogenic markers and compressive moduli of the injectable CMG-MSCs/alginate hydrogels incubated in chondrogenic medium were higher compared to the hMSCs directly encapsulated in alginate hydrogels.

## 1. Introduction

The elderly population, particularly those over the age of 60, experience joint pain due to cartilage degeneration which leads to long-term disability and lower quality of life [1]. The complex physical and biochemical properties of articular cartilage have resulted in limited clinical success in therapeutic intervention for patients with cartilage loss [2]. A promising approach is intervention with cellular therapies using autologous “adult human mesenchymal stem cells”, hereafter referred to as hMSCs. It has been shown that the bone marrow or synovium derived hMSCs delivered in a supportive matrix promote the expression of chondrogenic markers and produce a cartilage-like matrix in vitro and in vivo [3,4,5,6]. It is well-established that fetal articular cartilage has a higher capacity to regenerate the complex stratified structure of articular cartilage compared to the adult, which is attributed to the differences in extracellular matrix (ECM) composition between the adult and fetal articular cartilage [7,8]. Fetal articular cartilage has a higher fraction of Collagen IX (Col IX) and Col X and a lower fraction of Col II compared to those of the adult [9]. Novel engineering approaches are needed to recreate the structure and ECM composition of fetal articular cartilage to regenerate the injured tissue with cell therapy.

Synthetic and natural microcarriers are used as three-dimensional matrices in cell culture bioreactors for expansion of hMSCs as well as other stem cells [10,11,12,13,14,15,16]. In the case of synthetic microcarriers like polystyrene, polyvinyl alcohol, polydimethylsiloxane, polymethyl methacrylate, poly(2-oxazoline), polyacrylamide, polyethylene glycol, poly(lactide-co-glycolide), and their copolymers, an additional step is required after expansion to detach and separate the functional cells from the carrier for clinical applications because these carriers lack the natural chemical composition of the regenerating tissue [17]. Further, the persistence of nondegradable synthetic microbeads causes suboptimal tissue interconnectivity, leading to disintegration and mechanical failure of the regenerated tissue [17]. Natural and biomimetic microcarriers like collagen, gelatin, dextran, alginate, chitosan, chitin, and their derivatives do not require cell separation from the carrier, but these matrices cause undesirable phenotypic changes or untimely differentiation of the stem cells during expansion [18,19]. We hypothesized that a tissue-mimetic microcarrier based on decellularized fetal or adult bovine articular cartilage microgels, hereafter referred to as cartilage microgels (CMGs), could enhance the regenerative capacity of hMSCs for articular cartilage regeneration. The hMSCs expanded on fetal CMGs, hereafter referred to as fCMG-MSCs, could potentially mimic the process of fetal development of articular cartilage following injection in the joint capsule using minimally invasive arthroscopic techniques [20]. Here, we investigated the production of microgels from decellularized fetal or adult bovine articular cartilage and their use as microcarriers in the growth and expansion of hMSCs for use as injectable hydrogels for regeneration of human articular cartilage defects. The following approach was used. Decellularized, ground, and sorted adult or fetal bovine articular cartilage microgels (aCMGs or fCMGs) with average sizes of 90, 190, and 250 µm were used as microcarriers for growth and expansion of hMSCs (aCMG-MSCs or fCMG-MSCs). Next, the fCMG-MSCs or aCMG-MSCs were encapsulated in alginate hydrogels and crosslinked with calcium chloride to generate injectable cellular constructs (CMG-MSCs/Alg). The injectable constructs were incubated in chondrogenic medium and assessed with respect to compressive modulus and expressions of chondrogenic markers of the superficial zone (Sox-9 and SZP), middle zone (Col II and AGC), and calcified zone (Col X and ALP) of articular cartilage.

## 2. Results and Discussion

**Figure 1:**
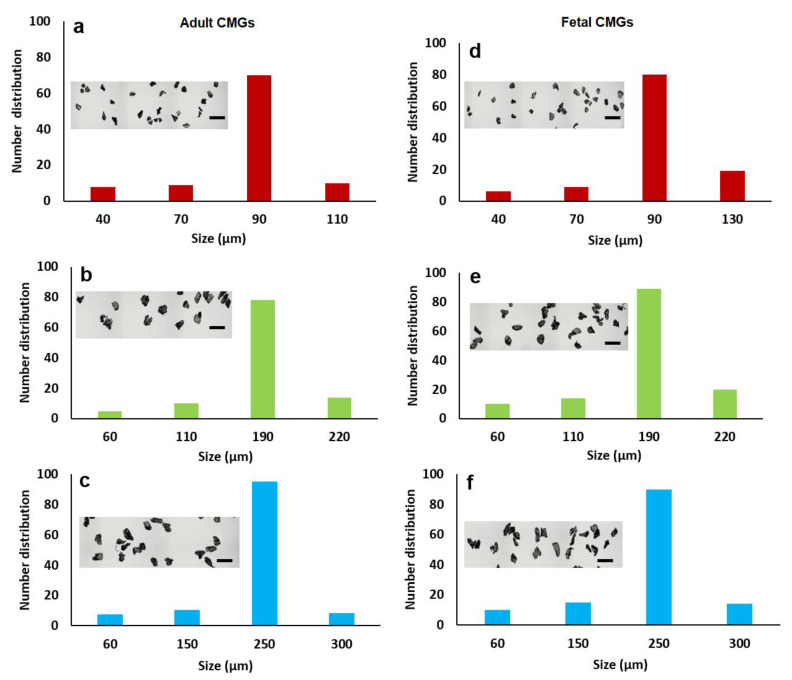
The size distribution of adult (**a**–**c**) and fetal (**d**–**f**) cartilage microgels (CMGs). The distributions (**a**–**c**) correspond to adult CMGs with average sizes of 90, 190, and 250 μm, respectively; distributions (**d**–**f**) correspond to fetal CMGs with average sizes of 90, 190, and 250 μm. The insets in the distributions are SEM images of the CMGs showing irregular shape of the microgels.

The size distribution of aCMGs and fCMGs with average sizes of 90, 190, and 250 µm are shown in Figure 1a–c and 1d–f, respectively; the insets in Figure 1a–f show the corresponding microscope images of the CMGs. The fraction corresponding to the average size was between 70 and 90% of the distribution for fetal and adult CMGs and all sizes. The fraction of microgels with a size greater than the average was higher for fCMGs compared to those for aCMGs, which was attributed to the more pliable nature of the former. The adult and fetal CMGs had irregular, nonspherical shapes.

The percent equilibrium water content and mass loss of fCMGs and aCMGs with average sizes of 90, 190, and 250 µm are shown in Table 1. For mass loss studies, the CMGs were incubated in PBS for 8 weeks. For all sizes, the water content of fCMGs was higher than that of aCMGs. The mass loss of fetal and adult CMGs increased with increasing size. For a given average size, the mass loss of aCMGs was slightly lower than that of fCMGs. The mass loss data indicated that the CMGs were stable with negligible hydrolytic degradation in the absence of enzymes.

**Figure 2:**
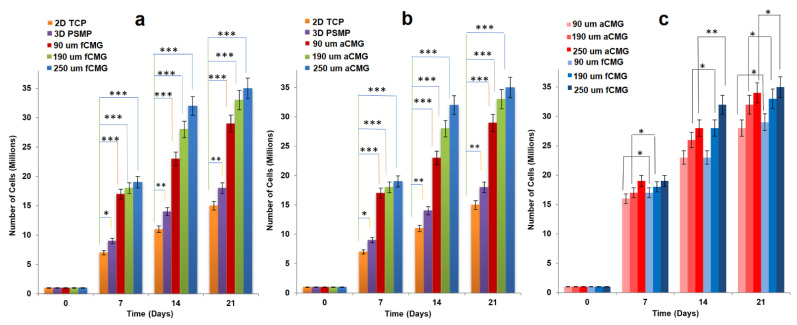
The growth of hMSCs on fetal (**a**) and adult (**b**) CMGs with culture time in basal medium in a tissue culture bioreactor for CMG sizes of 90 (red), 190 (green), and 250 μm (blue); hMSCs grown on 2D tissue culture plates (orange) and 3D polystyrene beads (purple) were used as controls; (**c**) comparison of hMSCs grown on fetal (blue) and adult (red) CMGs with culture time in basal medium for CMG sizes of 90 (light), 190 (medium), and 250 μm (dark); one asterisk (*), two asterisks (**), and three asterisks (***) indicate a statistically significant difference between two experimental groups at a given time point at the levels of *p* < 0.05, *p* < 0.01, and *p* < 0.001, respectively.

For cell expansion, CMGs were added periodically every two days to a suspension of hMSCs in basal culture medium. The periodic addition of CMGs allowed continuous expansion of hMSCs by the addition of more CMGs to the cell suspension with culture time as compared to the addition of all CMGs at time zero. Figure 2a,b compares the cell content of fetal and adult CMG-MSCs, respectively, having CMG average sizes of 90, 190, and 250 µm with hMSCs grown on solid polystyrene microparticles (PSMP) as a function of culture time. For all time points, the cell content of CMG-MSC groups was significantly higher (*p* < 0.001) than PSMP and 2D TCP controls (*p* < 0.001) and increased with culture time for all CMG sizes. The CMG-MSCs with 250 µm average size had the highest cell content compared to that of other sizes for fetal as well as adult CMG-MSCs. The lower cell content of the 90 µm CMG-MSCs was attributed to the higher inter-CMG cell transfer with each successive CMG addition to the culture medium. Inter-CMG transfer required cell detachment from a microgel, migration through the medium, and re-attachment to another microgel with a lower cell content, which increased the lag time between cell divisions. Figure 2c compares the cell contents of fetal and adult CMG-MSCs as a function of culture time for all average sizes. For a given time and average size, the cell content of fCMGs was significantly higher (*p* < 0.001) than that of aCMGs only for the 250 µm average size and time point of 14 days.

**Figure 3:**
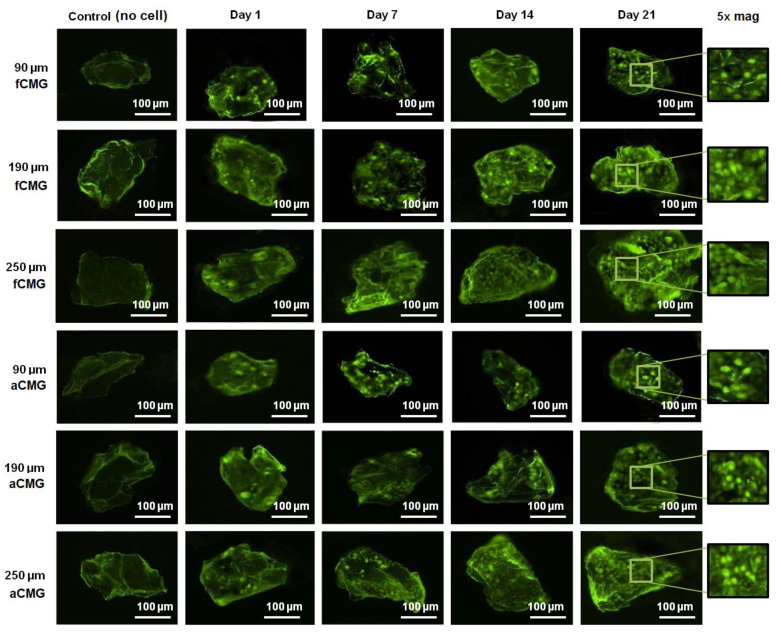
The fluorescent images of live (green) and dead (red) cells in a randomly selected microgel from fetal and adult CMG-MSCs in basal medium as a function of culture time (1, 7, 14, and 21 days) for CMG sizes of 90, 190, and 250 μm; the control group (leftmost column) was the image of a microgel without hMSCs incubated in basal medium with culture time; the images in the last column on the right re 5× magnification of the 21 days images. The scale bar in the images is 100 μm.

The fluorescent images of a randomly selected microgel in fetal and adult CMG-MSCs of different average sizes are shown in Figure 3 as a function of culture time. The green and red fluorescence in the images correspond to live and dead cells, respectively. The images showed the presence of hMSCs in the CMGs’ porous structure. The fluorescent intensity of live cells increased with culture time for fetal and adult CMG-MSCs and for all average sizes. After 21 days of culture, the live cell intensity of fCMG-MSCs was slightly higher than that of aCMG-MSCs, whereas the intensity was independent of CMGs’ average size. The fluorescent images showed >95% cell viability of hMSCs in CMG-MSCs.

**Figure 4:**
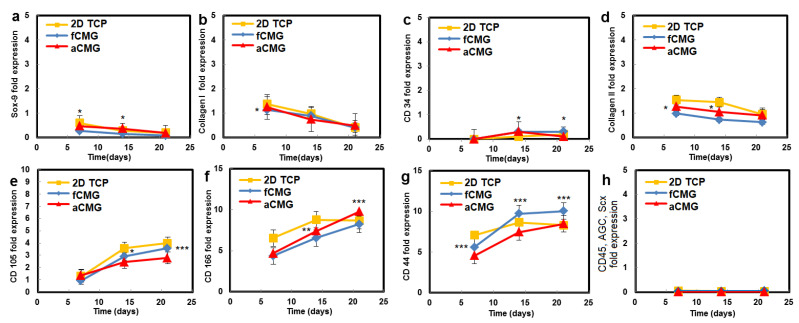
The mRNA expression of markers (**a**) Sox-9, (**b**) Col I, (**c**) CD34, (**d**) Col II, (**e**) CD105, (**f**) CD166, (**g**) CD44, and (**h**) CD45, AGC, Scx of hMSCs cultured on adult (red) and fetal (blue) CMGs in basal medium as a function of culture time; the control group (yellow) was hMSCs cultured on 2D tissue culture plates in basal medium. The average size of CMGs in CMG-MSCs for mRNA expression studies was 190 µm; one asterisk (*), two asterisks (**), and three asterisks (***) indicate a statistically significant difference between two experimental groups at a given time point at the levels of *p* < 0.05, *p* < 0.01, and *p* < 0.001, respectively.

The hMSCs used in this work had upregulated mRNA expressions of CD105, CD166, CD29, and CD44 markers and downregulated expressions of CD14, CD34, and CD45, consistent with the previously reported marker expressions [21,22]. Therefore, the hMSCs expanded on CMGs were evaluated with respect to the expressions of CD105, CD166, CD44, CD45, and CD34 markers (Figure 4). The average size of CMGs in CMG-MSCs for mRNA expression studies was 190 µm. The expressions of CD105, CD166, and CD44 markers of hMSCs expanded on fetal/adult CMGs in basal medium increased with time, whereas the expressions of CD45 and CD34 markers was downregulated for all time points, as shown in Figure 4. There was no difference in the expression of hMSC markers between adult and fetal CMG-MSCs. As the decellularized articular cartilage ECM can induce differentiation of hMSCs to the chondrogenic and osteogenic lineages, the expression of Sox-9 as the master regulator of chondrogenesis, Col II as the chondrogenic marker, and Col I as the osteogenic marker were measured with culture time [23], and the results are shown in Figure 4. The expressions of Sox-9, Col II, and Col I of CMG-MSCs decreased with culture time in basal medium. There was no difference in the expressions of chondrogenic markers of hMSCs between fCMG-MSCs and aCMG-MSCs. The results in Figure 4 demonstrate that hMSCs expanded on fetal or adult CMGs in basal medium maintained the expression of hMSC markers without premature differentiation.

**Figure 5:**
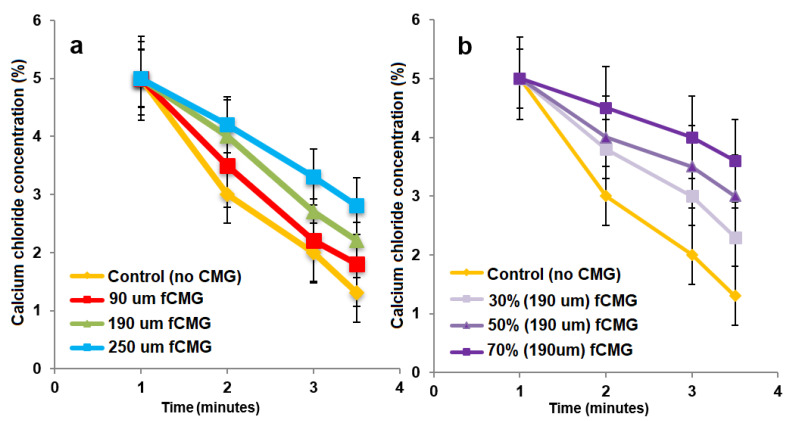
(**a**) The gelation time of alginate hydrogels as a function of CaCl_2_ concentration containing 50% CMGs (by alginate weight) with microgel sizes of 90 (red), 190 (green), and 250 μm (blue); (**b**) the gelation time of alginate hydrogels as a function of CaCl_2_ concentration containing 30% (very light purple), 50% (light purple) and 70% (purple) CMGs with an average size of 190 μm. The control group in (**a**,**b**) was alginate hydrogel without CMGs.

For the delivery of CMG-MSCs as an injectable hydrogel in treating articular cartilage defects, fetal or adult CMG-MSCs were suspended in an alginate precursor solution, the suspension was injected in a mold and crosslinked with CaCl_2_ as a chelating agent to generate aCMG-MSCs/Alg or fCMG-MSCs/Alg cellular constructs. The gelation of pristine alginate solution without CMGs was used as a control. The effect of CMG size on gelation time of the alginate solutions is shown in Figure 5a as a function of CaCl_2_ concentration. For a given CMG size, gelation time decreased with increasing CaCl_2_ concentration. For a given CaCl_2_ concentration, gelation time increased with increasing CMG size. The effect of CMG loading on gelation time of the alginate solutions is shown in Figure 5b as a function of CaCl_2_ concentration. For a given CaCl_2_ concentration, gelation time decreased with increasing CMG loading from 30% to 70%. Overall, gelation times were in the range of 1–4 min, which were within the clinically acceptable range for in situ gelling injectable constructs [24].

**Figure 6:**
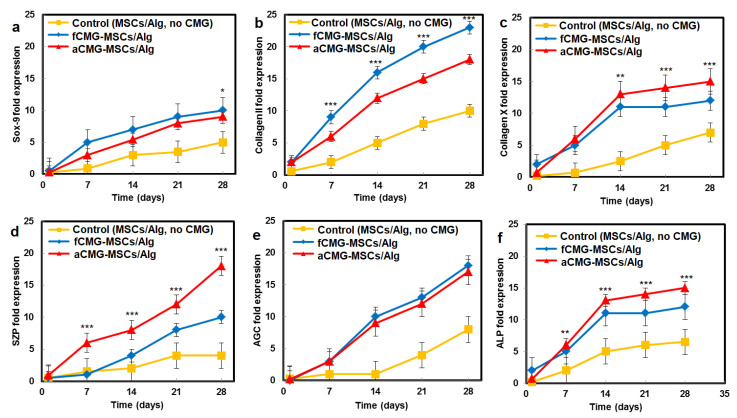
The mRNA expression of chondrogenic markers (**a**) Sox-9, (**b**) Col II, (**c**) Col X, (**d**) SZP, (**e**) AGC, and (**f**) ALP with incubation time for fCMG-MSCs (blue) and aCMG-MSCs (red) encapsulated in alginate hydrogels and cultured in chondrogenic medium for 28 days; the control group (yellow) was hMSCs (without CMGs) encapsulated in the alginate hydrogel and cultured in chondrogenic medium. The average size of CMGs of the hydrogel constructs for mRNA expression studies was 190 µm; one asterisk (*), two asterisks (**), and three asterisks (***) indicate a statistically significant dif-ference between two experimental groups at a given time point at the levels of *p* < 0.05, *p* < 0.01, and *p* < 0.001, respectively.

The mRNA expressions of chondrogenic markers for adult and fetal CMG-MSCs/Alg hydrogel constructs with culture time in chondrogenic medium are compared in Figure 6. The average size of CMGs of the hydrogel constructs for mRNA expression studies was 190 µm. The hMSCs encapsulated in the alginate gel without CMGs was used as the control group. The mRNA expressions included Sox-9 [23], SZP as the superficial zone marker [25], Col II, and AGC as the middle zone markers [26], and Col X and ALP as markers of cartilage hypertrophy in the calcified zone of articular cartilage [27]. For all markers and culture times, the expressions for aCMG-MSCs and fCMG-MSCs were higher than the control group. The aCMG-MSCs/Alg and fCMG-MSCs/Alg had similar expressions of Sox-9, AGC, Col X, and ALP markers. The expressions of SZP and Col II markers of aCMG-MSCs/Alg were higher and lower than those of fCMG-MSCs/Alg, respectively. The differentiation of hMSCs to zone-specific phenotypes is regulated by zone-specific growth factors in the culture medium as previously reported [26]. As the culture medium was not supplemented with zone-specific growth factors, a difference in the expressions of zone-specific markers between the adult and fetal CMG/MSCs/Alg was not expected. The marker expressions in Figure 6 demonstrate that adult and fetal CMGs enhanced chondrogenic differentiation of hMSCs in the hydrogel constructs as compared to that of the method of direct injection of hMSCs suspended in alginate hydrogels for the repair of articular cartilage defects [28].

**Figure 7:**
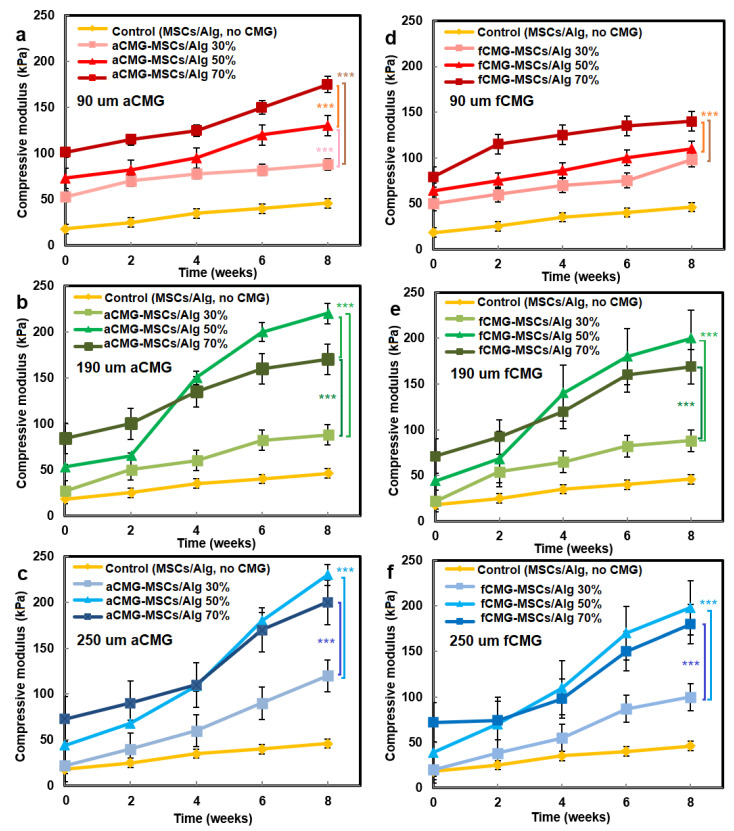
The compressive modulus of aCMG-MSCs/Alg (left column) and fCMG-MSCs/Alg (right column) hydrogels with culture time in chondrogenic medium as a function of CMG loading and average CMG size; CMG loadings were 30% (light shade), 50% (medium shade), and 70% (dark shade). The average CMG sizes in (**a**,**d**), (**b**,**e**), and (**c**,**f**) were 90 (**a**), 190 (**b**), and 250 μm (**c**), respectively; the control group (yellow) was MSCs (without CMGs) encapsulated in the alginate hydrogel and cultured in chondrogenic medium; three asterisks (***) indicate a statistically significant dif-ference between two experimental groups at a given time point at the level of *p* < 0.001.

The compressive modulus of aCMG-MSCs/Alg and fCMG-MSCs/Alg hydrogels was measured as a function of CMG loading with culture time in chondrogenic medium, and the results are shown in Figure 7a–f, respectively. The hMSCs encapsulated directly in the alginate hydrogel and cultured in chondrogenic medium were used as the control. For all adult and fetal CMG-MSCs/Alg hydrogel constructs, the modulus increased continuously with culture time. For any culture time, the modulus of adult and fetal CMG-MSCs/Alg constructs was higher than that of the control group (yellow curve) for all CMG loadings and sizes. The moduli of adult and fetal CMG-MSCs/Alg hydrogels with CMG loadings of 50% and 70% were higher than that with 30% CMG loading for all CMG sizes and incubation times. The moduli of adult and fetal CMG-MSCs/Alg hydrogels with CMG sizes of 190 and 250 μm were higher than that with 90 μm CMG size for all CMG loadings and culture times. For adult cartilage microgels, the CMG-MSCs/Alg hydrogel with 250 μm size and 50% CMG loading had highest modulus of 238 ± 10 kPa after eight weeks of incubation. For fetal cartilage microgels, the CMG-MSCs/Alg hydrogels with 250 or 190 μm size and 50% CMG loading had the highest modulus at 197 ± 20 kPa after eight weeks of incubation. Overall, the adult CMG-MSCs/Alg hydrogel with 250 μm size and 50% CMG loading had the highest modulus. As the extent of chondrogenic differentiation of hMSCs has been shown to increase with increasing cell–cell contact [29], the higher modulus of adult or fetal CMG-MSCs/Alg hydrogels with CMG sizes of 250 and 190 μm was attributed to the higher cell density per CMG, which increased cell–cell interaction and chondrogenesis.

**Figure 8:**
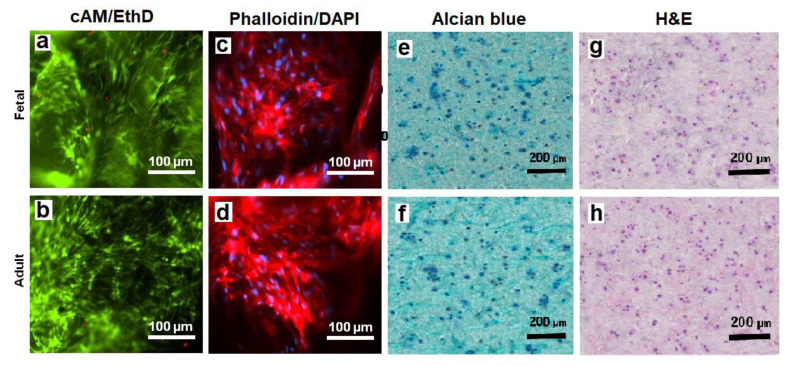
The images of sectioned fetal (first row) and adult (second row) CMG-MSCs/Alg hydrogels with cAM/EthD staining for live/dead cells (**a**,**b**), Phalloidin/DAPI staining for cell shape (**c**,**d**), Alcian blue staining for GAG (**e**,**f**), and H&E staining for cell distribution (**g**,**h**); the CMG loading in the hydrogel constructs was 50%, the CMGs average size was 190 μm, and the hydrogel constructs were cultured in chondrogenic medium for 21 days. The scale bar in cAM/EthD and Phalloidin/DAPI images is 100 µm; the scale bar in Alcian blue and H&E images is 200 µm.

The viability of hMSCs in CMG-MSCs/Alg hydrogels was imaged with live/dead cAM/EthD staining, and the cell shape was imaged with Phalloidin/DAPI staining. Figure 8a,b shows live/dead staining of the hMSCs in fetal and adult CMG-MSCs/Alg hydrogels, respectively, with 50% CMG loading, 190 μm CMG size after 21 days of culturing in chondrogenic medium. The live/dead images showed >99% viability of the hMSCs in fetal or adult CMG-MSCs/Alg. The Phalloidin/DAPI stains in Figure 8c,d shows the spindle-shaped hMSCs in fetal and adult CMG-MSCs/Alg hydrogels, respectively. Figure 8e,f shows Alcian blue stained sections of fetal and adult CMG-MSCs/Alg hydrogels, respectively, showing the accumulation of GAG in these sections. The CMG loading of the hydrogels in Figure 8e,f was 50%, and their average size was 190 μm. Figure 8g,h shows H&E-stained images of the above hydrogels showing uniform distribution of CMG-MSCs in the alginate matrix. The results in Figure 4, Figure 5, Figure 6, Figure 7 and Figure 8 demonstrated that hMSCs could be expanded on adult or fetal articular cartilage microgels (CMGs) while maintaining their MSC marker expression, the expanded cells could be encapsulated in alginate hydrogels, injected into a defect to form a crosslinked cellular hydrogel construct without the need to separate the cells from the microgel carrier, and the CMGs supported differentiation of CMG-MSCs to the chondrogenic lineage.

## 3. Conclusions

This work describes a novel approach to produce injectable hydrogels for delivery of hMSCs expanded on biomimetic microcarriers in regeneration of articular cartilage. First, the fetal or adult bovine articular cartilage was minced, decellularized, and freeze-dried. The freeze-dried extracellular matrix (ECM) was ground and sieved to produce articular cartilage microgels (CMGs) within a specified size range. Next, human MSCs were seeded on CMGs and expanded to generate CMG-MSCs. The expanded CMG-MSCs maintained their expression of mesenchymal markers in basal medium. Then, CMG-MSCs were suspended in sodium alginate, injected into a mold, crosslinked with calcium chloride, and incubated in chondrogenic medium as an injectable cellular construct for articular cartilage regeneration without the need to separate the expanded hMSCs from the microcarrier. The fetal or adult CMG-MSCs in alginate hydrogels showed significantly higher expression of chondrogenic markers as well as higher compressive modulus with culture time in chondrogenic medium compared to hMSCs directly encapsulated in alginate gels.

## 4. Materials and Methods

### 4.1. Production of Decellularized Bovine Articular Cartilage Microgels

Full-thickness articular cartilage harvested from fetal or adult bovine femoral condyles (Animal Technologies, Tyler, TX, USA) was decellularized as we described previously [5]. Briefly, the articular cartilage samples were dissected with a scalpel into small pieces, and the dissected pieces were frozen in liquid nitrogen and milled. The milled fragments were decellularized by immersion in 10 Mm Tris/1% triton solution for 24 h, followed by sonication for 2 h to form a microgel suspension. Next, the suspension was incubated in 1 U/mL deoxyribonuclease and 1 U/mL ribonuclease (Amresco, Dublin, Ireland) in phosphate buffer saline (PBS; Life Technologies, Grand Island, NY, USA) for 72 h at 37 °C to digest DNA and RNA [30]. Then, the suspension was centrifuged, the supernatant was discarded, and the precipitate was freeze-dried. The freeze-dried fragments were further ground (Hamilton Beach, Southern Pines, NC, USA) and sorted for size by progressively passing through sieves (Rosin Tech, Los Angeles, CA, USA) ranging in size from 80 to 300 µm to form 90 (40–110 µm size range), 190 (60–220 µm size range), and 250 µm (60–300 µm size range) cartilage microgels (CMGs). Adult and fetal CMGs are hereafter referred to as aCMGs and fCMGs, respectively.

### 4.2. Characterization of the Decellularized Articular Cartilage Microgels

The size distribution of CMGs was imaged with a light microscope. The captured 2D images were analyzed with ImageJ software (version 1.53n released 7 November 2021, National Institutes of Health, Bethesda, MD, USA) to determine the average size as we described previously [31]. The procedure for creating the size distribution data is as follows. Following grinding, the CMG powder was sieved sequentially using 300 μm, 250 μm, 190 μm, and 90 μm sieves. Particles retained on the 250 μm, 190 μm, and 90 μm sieves were collected and designated as the 250 μm, 190 μm, and 90 μm CMG fractions, respectively. The particles retained in each fraction were subsequently imaged using light microscopy and analyzed with ImageJ software. The software quantified the particle size distribution based on particle area, which was converted to equivalent circular diameter by assuming a two-dimensional circular projection of the particles. The bar charts shown in Figure 1 represent the number of particles as a function of equivalent circular diameter. Only particle sizes with a minimum count of four particles were included in the plots; therefore, the intervals between bars are not equally spaced. The distributions shown represent the raw particle count data obtained from ImageJ analysis. For example, the distribution of adult cartilage 190 μm CMGs shown in Figure 1b corresponds to particles that passed through the 250 μm sieve and were retained by the 190 μm sieve. The resulting distribution demonstrates that particles with an equivalent diameter of approximately 190 μm constituted the predominant fraction, while a smaller proportion of particles exhibited slightly lower or higher diameters. After size analysis, the dried CMGs with freshly cut surfaces were coated with gold using a Denton Desk II sputter coater (Moorestown, NJ, USA) and imaged for morphological analysis with a TESCAN VEGA3 SBU variable-pressure scanning electron microscope (SEM; Kohoutovice, Czech Republic).

The equilibrium fractional water content of CMGs was measured by incubation in PBS at 37 °C as we described previously [32]. Briefly after swelling in PBS, the CMG samples were filtered, unbound water was removed with a filter paper, and the weight of the filtered samples was measured. After weighing, the samples were returned to a fresh solution and incubated until the next time point. The equilibrium water content of the CMG samples was calculated as the difference between the initial and swollen weights divided by the total weight. The mass loss of the samples was measured at one time point after 8 weeks of incubation. After 8 weeks, the CMG samples were filtered, freeze-dried, and their weight was measured. Mass loss was defined as the difference between the initial and final weight of the dry CMGs divided by the initial weight.

### 4.3. Culture of hMSCs on Articular Cartilage Microgels

Adult MSCs harvested from healthy human bone marrow with high expressions of CD105, CD166, CD29, and CD44 and low expressions of CD14, CD34, CD45, and TGF-β1 markers (passages 3–5; Lonza, Allendale, NJ, USA) were expanded in basal medium (BM) consisting of high glucose DMEM (Life Technologies) supplemented with 10% fetal bovine serum (FBS; Life Technologies), 100 units/mL penicillin G (Sigma-Aldrich, St. Louis, MO, USA), and 100 µg/mL streptomycin (Sigma-Aldrich) as we previously described [5]. After sterilization with ethanol and ultraviolet (UV) radiation [33], the CMGs were incubated in basal medium overnight to reach equilibrium swelling prior to cell seeding. The initial seeding density was calculated based on the typical average size of hMSCs (18 µm average diameter [34]) and 20% CMG surface coverage. The CMGs were characterized by an average size equivalent to the diameter of a sphere with cubical pores. For 3D microcarrier cell culture, the specified amounts of CMGs were added to the basal medium in ultra-low attachment tissue culture flasks followed by the addition of 3 × 10^6^ hMSCs. The flasks were securely mounted on a rocker mixer and placed in a humidified 5% CO_2_ incubator at 37 °C. Based on previous reports, the doubling time of hMSCs was approximately 48 h [35]. For a given cell culture experiment, the cell density on CMGs was initially very low when all CMGs were added to the medium at time zero, which led to a long lag time for cell growth. Therefore, CMGs were added periodically, approximately every cell doubling time of 48 h in four batches, to maintain a relatively constant cell density on CMGs and avoid a long lag time. The analysis of images of hMSC seeded CMGs confirmed the absence of cell crowding. The hMSCs cultured on 2D adherent culture flasks or 3D polystyrene microbeads (50 µm average size; Advance Scientific, Moffat Beach, QLD, Australia) were used as controls. At each time point (7, 14, and 21 days), the cultures were characterized with respect to cell number, viability, and mRNA expression of hMSC markers. The hMSCs cultured on aCMGs and fCMGs are hereafter referred to as aCMG-MSCs and fCMG-MSCs, respectively.

### 4.4. Characterization of hMSCs Cultured on Articular Cartilage Microgels

At each time point (7, 14, and 21 days), 2 mL of 0.05% trypsin/0.53 mM EDTA (Life Technologies) was added to 1 mL of CMG-MSCs suspension and incubated for 15 min under shaking to detach hMSCs from the CMGs. Next, the suspension was transferred on a 40 µm nylon cell strainer (40 and 70 µm sizes, Corning, Canton, NY, USA) fixed on a 50 mL Falcon tube and washed with DMEM using an insulin syringe. The filtrate was centrifuged at 400× *g* for 5 min, and the separated cells were counted with a hemocytometer as we described previously [36]. For cell viability, the CMG-MSCs were incubated with 1 µg/mL acetomethoxy derivative of calcein and ethidium homodimer (cAM/EthD; Life Technologies) to stain live and dead cells, respectively, as we described previously [32]. The stained cells were imaged using an inverted fluorescent microscope (Nikon Eclipse Ti-e, Nikon, Melville, NY, USA).

The CMG-MSCs were characterized phenotypically by their mRNA expressions of CD105, CD166, CD44, CD45, and CD34 markers as reported previously [37]. Further, the CMG-MSCs were tested for differentiation to the chondrogenic lineage by measuring the mRNA expressions of chondrogenic markers, Sox-9, Collagen I (Col I), Col II, and aggrecan (AGC) [5,6]. At each time point, hMSCs were separated from CMGs, and the total RNA of the homogenized cell suspension was isolated using TRIzol as we described previously [31]. The genomic DNA was removed using deoxyribonuclease I (Invitrogen) as previously described [26]. Of the extracted RNA, 250 ng, as measured using a Nanodrop spectrophotometer, was converted to cDNA using a reverse transcription system (Promega, Madison, WI, USA). The cDNA was amplified with SYBR green RealMasterMix (Eppendorf, Hamburg, Germany) using a Bio-Rad CXF96 real-time quantitative polymerase chain reaction system (rt-qPCR; Hercules, CA, USA) and the appropriate gene-specific primers as described [6]. All forward and reverse primers were designed using Primer3 web-based software (version 0.4.0) as described [26] and synthesized by Integrated DNA Technologies (Coralville, IA, USA). The expressions were normalized against GAPDH reference gene, and fold changes were compared based on ∆∆ct method to those in the same group at day zero as previously described [38].

### 4.5. Encapsulation of CMG-MSCs in Alginate Hydrogel

CMG-MSCs were encapsulated in a sodium alginate hydrogel using the following procedure [39]. Solutions of 3 wt% sodium alginate (Ward’s Science, Henrietta, NY, USA) in PBS (100 mL) and 1 wt% CaCl_2_ in PBS (100 mL) were prepared and sterilized as described [33]. Suspensions of adult or fetal CMG-MSCs in culture medium with average CMG sizes of 90, 190, and 250 µm were prepared as described in the previous sections. The suspension was transferred to a sterilized Falcon tube, centrifuged, the medium was removed, and the alginate solution was added to the CMG-MSCs and mixed with a sterile glass rod. The surface of a sterile disk-shaped, Teflon mold with effective diameter of 2 cm and height of 1.5 mm was sprayed with the CaCl_2_ solution using an insulin syringe. Next, the suspension of CMG-MSCs in alginate was transferred to the mold, and the CaCl_2_ solution was sprayed on the exposed surface to crosslink the suspension. The gelation time was determined visually by placing a drop of the alginate solution on a glass surface followed by spraying the CaCl_2_ solution on the drop, tilting the surface by 45 degrees, observing the flow of the drop on the tilted surface, and recording the time for the drop to stop flowing as the gelation time. The crosslinking time of the hydrogel ranged from 1 to 4 min. After crosslinking, the hydrogel was removed from the mold, transferred to a petri dish, and cultured in chondrogenic medium for up to 8 weeks. Based on the mold used, the hydrogels were disk-shaped with diameter and thickness of 2 cm and 1.5 mm, respectively. The chondrogenic medium consisted of DMEM (4.5 g/mL glucose, 50 μg/mL l-proline, 50 μg/mL ascorbic acid, 0.1 mM sodium pyruvate, 1% *v*/*v* insulin-transferrin-selenium premix) supplemented with the 10 ng/mL TGF-β1 [26]. The hMSCs encapsulated directly in the alginate gel without CMGs were used as the control group. The aCMG-MSCs and fCMG-MSCs encapsulated in alginate gels are hereafter referred to as aCMG-MSCs/Alg and fCMG-MSCs/Alg, respectively.

### 4.6. Analysis of CMG-MSCs Encapsulated in Alginate Hydrogel

At each time point, adult or fetal CMG-MSCs/Alg were assessed with respect to compressive modulus, cellularity, and the expressions of chondrogenic markers Sox-9, Col II, AGC, the superficial zone marker SZP, and the calcified zone markers Col X and alkaline phosphatase (ALP). For cell viability, the samples were incubated with cAM/EthD live/dead stains, and the stained samples were imaged using the Eclipse Ti-E inverted fluorescent microscope as we described previously [26]. For cell cytoskeleton and nuclei staining, the samples were fixed with 4% paraformaldehyde, permeabilized using PBS containing 0.1% Triton X-100 for 5 min, and incubated with Alexa 488 phalloidin and DAPI as previously described [40]. The stained samples were imaged with a Nikon inverted fluorescent microscope. At each time point, the samples were homogenized, and the mRNA expressions of chondrogenic markers Sox-9, Col II, Col X, SZP, AGC, and ALP were measured as described for CMG-MSCs in previous sections. The CMG-MSCs/Alg hydrogels after 21 days of culture were analyzed histologically for cellularity and expression of GAG as we previously described [26]. Briefly, the samples were fixed in formalin, embedded in paraffin, and cryo-sectioned to a thickness of 10 µm. The sections were divided into two groups with the first group stained with hematoxylin and eosin-Y (H&E; Sigma-Aldrich) to ascertain distribution and morphology of the encapsulated cells, and Alcian blue (Sigma-Aldrich) to image GAG accumulation. The stained sections were imaged with a Nikon Optiphot Epi-fluorescent microscope.

### 4.7. Compressive Modulus of CMG-MSCs Encapsulated in Alginate Hydrogel

At each time point, adult or fetal CMG-MSCs/Alg hydrogels were loaded on the Peltier plate of an AR 2000ex rheometer (TA Instruments, New Castle, DE, USA) and subjected to a uniaxial compressive strain as we previously described [41]. A strain sweep from 0.01% to 500% strain at 10 Hz was performed to find the yield strain. Similarly, a frequency sweep from 0.01 to 100 Hz at 0.2% strain was performed to find the crossover frequency. A sinusoidal shear strain with a frequency above the crossover frequency and a strain amplitude below the yield strain was exerted on the sample, and the storage (G’) and loss moduli (G”) were recorded with time. The slope of the linear fit to the stress-strain curve for strains of <10% was taken as the compressive modulus of the sample.

### 4.8. Statistical Analysis

All experiments were done in triplicate, and quantitative data were expressed as means ± standard deviation. Significant differences between groups were evaluated using a two-way ANOVA with a replication test and two-tailed Student’s *t*-tests. A value of *p* > 0.05 was generally used for testing significant differences between experimental groups, unless otherwise specified.

## Figures and Tables

**Table 1:** Equilibrium water content and mass loss of fCMGs and aCMGs for CMG average sizes of 90, 190, and 250 µm.

EWC/Mass Loss Versus CMG Size	Equilibrium Water Content (%)		Mass Loss (%)	
	fCMGs	aCMGs	fCMGs	aCMGs
90 µm CMGs	18.6 ± 0.9	15.3 ± 1.5	4.4 ± 0.2	3.7 ± 0.3
190 µm CMGs	19.6 ± 0.9	16.5 ± 0.6	5.2 ± 0.2	4.3 ± 1.3
250 µm CMGs	20.0 ± 1.5	18.0 ± 0.8	6.5 ± 0.1	5.8 ± 0.5

## Data Availability

The data presented in this study are available on request from the corresponding author. The data are not publicly available due to protection of intellectual property.
