# Correction: Jabbari et al. Decellularized Articular Cartilage Microgels as Microcarriers for Expansion of Mesenchymal Stem Cells. *Gels* 2022, *8*, 148

**DOI:** 10.3390/gels12070621

**Published:** 2026-07-10

**Authors:** Esmaiel Jabbari, Azadeh Sepahvandi, Safaa Ibrahim Kader, Victor Anthony Madormo

**Affiliations:** 1 Biomaterials and Tissue Engineering Laboratory, Department of Chemical Engineering, University of South Carolina, Columbia, SC 29208, USA; sepahvan@mailbox.sc.edu (A.S.); kaders@mailbox.sc.edu (S.I.K.); victor_madormo@med.unc.edu (V.A.M.); 2 Department of Chemistry and Biochemistry, University of South Carolina, Columbia, SC 29208, USA

In the original publication [1], additional clarification regarding the particle size distribution analysis presented in Figure 1 was omitted from Section 4.2, “Characterization of the Decellularized Articular Cartilage Microgels”. The additional information should be added after the second sentence in Section 4.2, Paragraph 1 and the corrected text appears below.

“The size distribution of CMGs was imaged with a light microscope. The captured 2D images were analyzed with ImageJ software (version 1.53n released 7 November 2021, National Institutes of Health, Bethesda, MD, USA) to determine the average size as we described previously [31]. The procedure for creating the size distribution data is as follows. Following grinding, the CMG powder was sieved sequentially using 300 μm, 250 μm, 190 μm, and 90 μm sieves. Particles retained on the 250 μm, 190 μm, and 90 μm sieves were collected and designated as the 250 μm, 190 μm, and 90 μm CMG fractions, respectively. The particles retained in each fraction were subsequently imaged using light microscopy and analyzed with ImageJ software. The software quantified the particle size distribution based on particle area, which was converted to equivalent circular diameter by assuming a two-dimensional circular projection of the particles. The bar charts shown in Figure 1 represent the number of particles as a function of equivalent circular diameter. Only particle sizes with a minimum count of four particles were included in the plots; therefore, the intervals between bars are not equally spaced. The distributions shown represent the raw particle count data obtained from ImageJ analysis. For example, the distribution of adult cartilage 190 μm CMGs shown in Figure 1b corresponds to particles that passed through the 250 μm sieve and were retained by the 190 μm sieve. The resulting distribution demonstrates that particles with an equivalent diameter of approximately 190 μm constituted the predominant fraction, while a smaller proportion of particles exhibited slightly lower or higher diameters. After size analysis, the dried CMGs with freshly cut surfaces were coated with gold using a Denton Desk II sputter coater (Moorestown, NJ, USA) and imaged for morphological analysis with a TESCAN VEGA3 SBU variable-pressure scanning electron microscope (SEM; Kohoutovice, Czech Republic).”

In the original publication [1], three contributors listed as authors in the BioRxiv preprint version were not included in the published article. As a result, Safaa Ibrahim Kader and Victor Anthony Madormo have been added to the list of authors. The third individual was contacted and given the opportunity to be included as an author but declined authorship.

Accordingly, the author list has been corrected as follows:

Esmaiel Jabbari ^1,^*, Azadeh Sepahvandi ^1^, Safaa Ibrahim Kader ^1,2^ and Victor Anthony Madormo ^1^

^1^ Biomaterials and Tissue Engineering Laboratory, Department of Chemical Engineering, University of South Carolina, Columbia, SC 29208, USA

^2^ Department of Chemistry and Biochemistry, University of South Carolina, Columbia, SC 29208, USA

The corrected Author Contributions statement appears here:

Author Contributions: Conceptualization, E.J.; methodology, E.J. and A.S.; software, E.J. and A.S.; validation, E.J. and A.S.; formal analysis, E.J., A.S. and S.I.K.; investigation, E.J. and A.S.; resources, E.J.; data curation, E.J., A.S. and V.A.M.; writing—original draft preparation, E.J.; writing—review and editing, E.J. and A.S.; visualization, E.J. and A.S.; supervision, E.J.; project administration, E.J.; funding acquisition, E.J. All authors have read and agreed to the published version of the manuscript.

This clarification has been added to improve transparency regarding the methodology used to generate the particle size distributions shown in Figure 1 and to make the authorship consistent with the authorship presented in the preprint version of the manuscript deposited in the BioRxiv repository.

The authors state that the scientific conclusions are unaffected. This correction was approved by the Academic Editor. The original publication has also been updated.
